# Health policy and systems research: the future of the field

**DOI:** 10.1186/s12961-018-0359-0

**Published:** 2018-08-22

**Authors:** David H. Peters

**Affiliations:** Department of International Health, Johns Hopkins University, Bloomberg School of Public Health, 615 N. Wolfe St, Suite E-8527, Baltimore, MD 21205 United States of America

**Keywords:** Health policy, Health systems, Health systems research, Sustainable Development Goals

## Abstract

Health policy and systems research (HPSR) has changed considerably over the last 20 years, but its main purpose remains to inform and influence health policies and systems. Whereas goals that underpin health systems have endured – such as a focus on health equity – contexts and priorities change, research methods progress, and health organisations continue to learn and adapt, in part by using HPSR. For HPSR to remain relevant, its practitioners need to re-think how health systems are conceptualised, to keep up with rapid changes in how we diagnose and manage disease and use information, and consider factors affecting people’s health that go well beyond healthcare systems. The Sustainable Development Goals (SDGs) represent a shifting paradigm in human development by seeking convergence across sectors. They also offer an opportunity for HPSR to play a larger role, given its pioneering work on applying systems thinking to health, its focus on health equity, and the strength of its multi-disciplinary approaches that make it a good fit for the SDG era.

Globally, population health is being challenged in different ways, from climate change and growing air pollution and toxic environmental exposure to food insecurity, massive population migration and refugee crises, to emerging and re-emerging diseases. Each of these trends reinforce each other and concentrate their harms on the most vulnerable populations. Multi-level governance, together with novel regulatory strategies and socially oriented investments, are key to successful action against many of the new challenges, with HPSR guiding their design and evolution.

The HPSR community cannot be complacent about its successful, yet short, history. Tensions remain about how different stakeholders use HPSR such as the contrast between embedding research within government institutions versus independently evaluating and holding decision-makers accountable. Such tensions are inevitable in the boundary-spanning field that HPSR has become. We should strive to enhance the influence of HPSR by staying relevant in a changing world and embracing the strength of our diversity of disciplines, the range of problems addressed, and the opportunity of the SDGs to ensure that health and social benefits are more inclusive for people within and across countries.

In 1962, Burnet, the Nobel prize-winning immunologist, wrote that the twentieth century would be witness to “*the virtual elimination of infectious disease as a significant factor in social life*” [1] – a reminder to be humble when predicting the future effects of health research. Nonetheless, the last 20 years has brought impressive change in the growth of health policy and systems research (HPSR), and the settings in which it is applied. One safe prediction is that the HPSR landscape will continue to change and grow in complexity.

## The value of HPSR

The central idea behind HPSR is that research should inform and influence policies and systems to pursue health goals [2]. Health systems goals and the values that underpin them are enduring and should continue to be examined through HPSR. Contexts will change and new challenges will emerge, but research will still be needed to inform how to achieve the multiple health systems goals – improving effectiveness, equity and efficiency, expanding health services coverage, and enhancing people’s financial protection, while minimising costs and improving accountability and trust. HPSR provides the tools for Ministries of Health and other health organisations to become learning organisations, serving to lead and adapt to changes in the health sector.

## Re-thinking health systems in a changing context

The changing context will also challenge how we think about health systems; HPSR should have a central role in understanding change and how to intervene. The social, political and environmental conditions for healthy living are rapidly shifting, as are expectations about the role of the state, civil society and business. Information communications and other technologies are transforming the diagnosis and management of disease, as well as the collection, analysis and sharing of individual and population health data. Additionally, there are growing population pressures due to environmental degradation, urbanisation and aging along with new threats due to emerging diseases and the failure of poorly organised market systems for health services, technologies and financial products. Each condition is both a driver of change and an effect of another; they are interdependent issues in an increasingly interconnected world.

The Sustainable Development Goals (SDGs) represent a shifting paradigm in human development, moving from the building up of individual core sectors within countries to seeking convergence across co-influencing and co-dependent sectors. In a world where wealth inequality is escalating, the SDGs mark a shift from efforts to provide overall benefits to a nation to focusing on inclusive growth and tackling inequities as the core of development efforts. HPSR is well placed to take on these issues of the future since it has a traditional focus on understanding and addressing different types of disadvantage and inequity, taking advantage of various disciplines and approaches to address inequities, including social epidemiology, economics, participatory action research and ethics [3]. Further, HPSR has pioneered the application of systems thinking in health, providing a wide set of theories, frameworks and tools to examine and test how different elements of systems – actors, functions and their relationships – fit together to make an overall whole [4].

Efforts to strengthen health systems have been both facilitated and constrained by the dominance of the Health Systems Building Blocks model [5]. The model focuses on inputs and selected functions of a healthcare system, but was designed as a communication tool to indicate options for government investment, and not as an analytic or explanatory model of a complete health system. The building blocks model has especially neglected people (indeed the entire demand side of a health system) and institutions, the importance of dynamic linkages between stakeholders and functions in a health system, and connections between health systems and other related systems (e.g. education, economic development, ecology, etc.). To apply research to questions concerned with the linkages across sectors – as envisioned by the SDGs – it will be more important to consider the roles of people (as individuals, families, communities and larger populations) and the dynamic connections between policies and systems that affect people both inside the traditional health sector and through related sectors.

## Growing challenges for HPSR

There are many new issues and evolving roles for different stakeholders in a health system, as well as novel ways in which we can study and influence health systems. Globally, population health is being challenged in different ways. Ambient air pollution in cities and indoor air pollution in rural homes have become important risk factors for chronic diseases. Food insecurity is again a critical public concern, as climate change is projected to decrease crop yields, particularly in South America, Africa, South Asia and Australia, while contributing to increased food price volatility [6]. Poor nutrition, exposure to environmental toxins and a resurgence of vector-borne diseases, such as malaria and dengue, are all consequences of environmental degradation. The poor are especially vulnerable, as they are most exposed to the direct and indirect shocks of environmental degradation, are more vulnerable because they lose relatively more wealth, and are less resilient because they do not have the financial and social safety nets required to manage them and recover [7].

The growing phenomenon of antimicrobial resistance is another major threat to global health that needs to be tackled in both the health and agricultural sectors at local, national and global levels. The failure to develop new antimicrobials or ensure equitable access to existing antibiotics, while counterfeit and substandard drugs flourish, represent major market failures [8, 9]. New regulatory strategies, socially oriented investment and a realignment of incentives are needed at all levels. Multi-level governance is the key for successful action in containment strategies, supported by HPSR to assess how well they work and guide their evolution.

Population migration is another major social, political and health systems challenge. One billion individuals are now on the move globally, one-quarter of whom are crossing national borders. The estimated refugee population reached an unprecedented 19.6 million individuals worldwide in 2015, half of whom are children [10]. Health systems are at the forefront of the response to the ongoing crisis facing refugees and other migrants, both at first point of contact and later during resettlement. There is a need to develop more effective approaches that respond to the health needs of displaced populations, yet the evidence base regarding which interventions are effective is quite weak.

## Opportunities for HPSR

HPSR has developed as a boundary-spanning field, not only crossing disciplinary lines, but also linking stakeholders with very different roles (e.g. policy-makers, health practitioners, researchers, civil society leaders, the media). As such, HPSR should continue to influence policy both within and across countries. HPSR has served in each of six types of research utilisation as described by Weiss [11], though in recent years it has tended to be used most directly in a problem-solving model (to facilitate decisions by policy-makers and managers) or to otherwise contribute to complex policy-making through an interactive model of health research. However, there is also a growing tension between new approaches that promote embedded and implementer-led research, which pursues problem-solving from ‘within’ [12, 13] and research that takes an external perspective, seeking to independently evaluate policy effects, identify neglected problems or hold decision-makers accountable [14, 15]. HPSR should be used to serve each of these perspectives, and not become captive to a single approach.

Encouraging diversity and equity has become part of the shared values of many practitioners and users of HPSR, crossing contexts and types of research utilisation. For example, the Alliance for Health Policy and Systems Research, along with many partners working in global health, have expressed a very clear set of values – “*to address problems of inequity, poverty and disadvantage*” [16] and to support partnerships and collaboration on an inclusive and participatory basis.

However, this set of values has come into conflict with recent policy and electoral decisions made around the world over the last few years. There has been a rise in electoral trends that seem to undermine the values of global citizenship and even the role of evidence in decision-making. Recent political events around the world, including in the Americas, Europe, Africa, and Asia, suggest that massive numbers of people are dissatisfied with incumbent policy-makers and their policies, and are voting for the politics of division. In contrast, HPSR can be a vehicle to learn from and promote the diversity of cultures, building of local capabilities and forging of international cooperation. The promise of research and its application to policy and public health practice can help people to overcome the divisions of nationalism, race, class, wealth and other obstacles to social justice and health equity.

## Conclusions

This is a time when the technical skills, knowledge contributions and historical values of HPSR are needed more than ever. We need a HPSR agenda to better understand and meet peoples’ expectations, and to sharpen our science of communication, both issues within the remit of HPSR. However, the HPSR community cannot be complacent about its successful but short-lived history. We should strive to enhance the influence of HPSR by staying relevant in a changing world, embracing the strength of our diversity of disciplines, the range of problems addressed, and the opportunity of the SDGs to ensure that health and social benefits are more inclusive for people within and across countries. If we are to have policies and interventions that promote justice and good health whilst being grounded in evidence, then we must ensure that our thinking and practice of HPSR help us rise to these challenges.

## Abbreviations

HPSR: health policy and systems research
SDGs: Sustainable Development Goals

## References

[1] Burnet FM (1962). Natural History of Infectious Disease.

[2] Alliance for Health Policy and Systems Research (2017). World Report on Health Policy and Systems Research.

[3] Gilson L (2012). Health Policy and Systems Research: A Methodology Reader.

[4] Peters DH (2014). The application of systems thinking in health: why use systems thinking?. Health Res Policy Syst..

[5] World Health Organization (2007). Everybody Business: Strengthening Health Systems to Improve Health Outcomes - WHO’s Framework for Action.

[6] Havlik P, Valin H, Gusti M, Schmid E, Leclère D, Forsell N, Herrero M, Khabarov N, Mosnier A, Cantele M, Obersteiner M. Climate change impacts and mitigation in the developing world: an integrated assessment of the agriculture and forestry sectors. Policy Research Working Paper No. WPS 7477. 2015. http://pure.iiasa.ac.at/11657. Accessed 14 Aug 2018.

[7] Hallegatte S, Bangalore M, Bonzanigo L, Fay M, Kane T, Narloch U, Rozenberg J, Treguer D, Vogt-Schilb A (2016). Shock Waves: Managing the Impacts of Climate Change on Poverty. Climate Change and Development.

[8] Cars O, Högberg LD, Murray M, Nordberg O, Sivaraman S, Lundborg CS, So AD, Tomson G (2008). Meeting the challenge of antibiotic resistance. BMJ.

[9] Peters DH, Bloom G (2012). Developing world: bring order to unregulated health markets. Nature.

[10] United Nations Population Division. International Migrant Stock 2015. 2016. http://www.un.org/en/development/desa/population/migration/data/estimates2/estimates15.shtml. Accessed 14 Aug 2018.

[11] Weiss CH (1979). The many meanings of research utilization. Public Adm Rev..

[12] MacGregor H, Bloom G (2016). Health systems research in a complex and rapidly changing context: ethical implications of major health systems change at scale. Dev World Bioeth.

[13] Tran N, Langlois EV, Reveiz L, Varallyay I, Elias V, Mancuso A (2017). Embedding research to improve program implementation in Latin America and the Caribbean. Rev Panam Salud Publica.

[14] Gaventa J, McGee R (2013). The impact of transparency and accountability initiatives. Dev Policy Rev.

[15] World Health Organization (2011). Monitoring, Evaluation and Review of National Health Strategies: A Country-led Platform for Information and Accountability.

[16] Alliance for Health Policy and Systems Research (2016). Investing in Knowledge for Resilient Health Systems: Strategic Plan 2016–2020.

